# Intravitreal Bevacizumab nanoparticles ameliorates retinal vasculopathy in an in vivo mouse model of retinopathy of prematurity

**DOI:** 10.1038/s41390-025-04508-w

**Published:** 2025-10-27

**Authors:** Suganesh Raghunathan, Ben Amadi, Alfiya Raja, Olachi J. Mezu-Ndubuisi

**Affiliations:** 1 Department of Pediatrics, University of Rochester School of Medicine and Dentistry, Rochester, NY USA; 2 Department of Ophthalmology, University of Rochester School of Medicine and Dentistry, Rochester, NY USA

## Abstract

**Background:**

Nanoparticles enable sustained drug release. Infants with retinopathy of prematurity (ROP) treated with Bevacizumab, an anti-vascular endothelial growth factor monoclonal antibody, develop systemic toxicity due to repeat dosing from a short half-life. We optimized fabrication of Bevacizumab nanoparticles in a mouse oxygen-induced retinopathy (OIR) model.

**Methods:**

Bevacizumab nanoparticle fabrication was optimized for polymer type and ratio, solvent, drug loading capacity (DL) %, and encapsulation efficiency (EE) %. Nanoparticles were characterized using Scanning electron microscopy (SEM), Transmission electron microscopy (TEM), and Fourier-transform infrared spectroscopy (FTIR). C57BL6/J mice exposed to OIR received intravitreal Empty (*n* = 18), Bevacizumab-loaded nanoparticles (*n* = 18), or Free Bevacizumab (*n* = 12) at P13 to right eyes. Fluorescein Angiography (FA) was performed at P20. Retinal avascular area, arterial tortuosity, and vein width were quantified.

**Results:**

SEM showed spherical nanospheres. TEM and FTIR confirmed drugs within nanospheres. DL and EE were optimized at 2.3% and 80%. There was sustained drug release of 80% at 72 h. FA showed increased retinal avascular area, reduced retinal artery tortuosity, and retinal vein width in Bevacizumab nanoparticle treated right eyes.

**Conclusions:**

Intravitreal administration of Bevacizumab-loaded nanoparticles significantly improved retinal vasculopathy in mice with OIR, without repeat dosing. Further toxicity studies are needed before clinical translation.

**Impact:**

- Retinopathy of Prematurity is treated with anti-vascular endothelial growth factor drugs such as Bevacizumab.
- Anti-VEGF drugs result in systemic toxicity from repeat dosing due to their short half-life.
- We, for the first time, show the feasibility and optimization of nanoparticle formulation of bevacizumab in a mouse model of ROP.
- Intravitreal Bevacizumab nanoparticles may prevent systemic toxicity and improve outcomes in infants with ROP.

## Introduction

Retinopathy of Prematurity (ROP) is a disorder of abnormal retinal vascularization, structure, and function in premature infants exposed to supplemental oxygen.^1–3^ Despite current treatment, ROP remains the leading cause of childhood blindness worldwide.^4–7^ The International Classification of Retinopathy of Prematurity (IC-ROP) defines ROP in Stages, characterizing the severity of abnormal retinal vascularization, and Zones, specifying the location of abnormal vascularization relative to the optic disc.^8^ Retinal artery tortuosity and retinal vein dilation signify plus disease, a marker of severe ROP,^1,9^ and can be objectively quantified in vivo in a mouse model of oxygen-induced retinopathy (OIR).^1,2^ Bevacizumab (Beva), a recombinant humanized monoclonal antibody that non-selectively blocks vascular endothelial growth factor (VEGF) isoforms is used as first line or adjunct therapy for pathologic neovascularization from ROP.^10–13^ Bevacizumab is limited in efficacy due to a short half-life,^14^ requiring repeat injections to maintain therapeutic efficacy, leading to systemic organ toxicity, retinal detachment, and a high rate of recurrence or reactivation of neovascularization.^11,15–19^ Administration of a sustained release formulation of Bevacizumab rather than free unencapsulated Bevacizumab has the potential to improve drug bioavailability and reduce the need for repeat treatments and systemic toxicity.^20,21^

Biodegradable polymers are utilized in medical engineering, biomedicine, and nanomedicine to optimize drug delivery and reduce toxicity. Poly -D, L-lactide-co-glycolic acid (PLGA), a co-polymer of lactic and glycolic acid, is one of the most successfully used polymers because it is hydrolyzed in the body to biodegradable, relatively non-toxic metabolite monomers, lactic acid and glycolic acid. Nanoparticles formed from polymers can have drugs incorporated, either conjugated to the surface of the nanoparticles or encapsulated within the core of the nanoparticle, where it is protected.^22^ We have previously demonstrated that intravitreal VEGFA_165a_-loaded PLGA microparticles promoted earlier retinal vascularization in mice with OIR.^23^ Nanoparticles are smaller than microparticles, and capable of deeper tissue penetration and more bioavailability.

We sought to increase the bioavailability of intravitreal Bevacizumab nanoparticles to reduce repeat dosing and systemic toxicity, and compare the efficacy of free versus nanoparticle Bevacizumab in ameliorating retinal vasculopathy in mice with OIR. Bevacizumab has been encapsulated into PLGA nanoparticles, with limitations in bioavailability.^20,24^ Polyvinyl Alcohol (PVA) is a non-toxic, hydrophilic polymer that facilitates drug encapsulation and stability, currently approved for devices such as contact lenses and artificial organs.^25,26^ PVA and PLGA-based polymeric nanoparticles encapsulate hydrophilic and lipophilic drugs for targeted and sustained release.^27,28^ We optimized the fabrication of Bevacizumab in a PVA-PLGA nanoparticle by varying the drug: polymer ratio and solvent prior to testing its efficacy with intravitreal delivery in a mouse OIR model of retinopathy of prematurity.

## Methods

### Animals

All studies were approved by the University Committee on Animal Resources of University of Rochester. Eight cages of C57BL6/J mice (Jackson Lab, Bar Harbor, ME) were allocated to Free Bevacizumab (3 cages, *n* = 18), Empty PLGA Nanoparticle (3 cages, *n* = 18), and Bevacizumab-loaded Nanoparticle (2 cages, *n* = 12) groups.

### In vivo OIR mouse model of retinopathy of prematurity

**Fig. 1:**
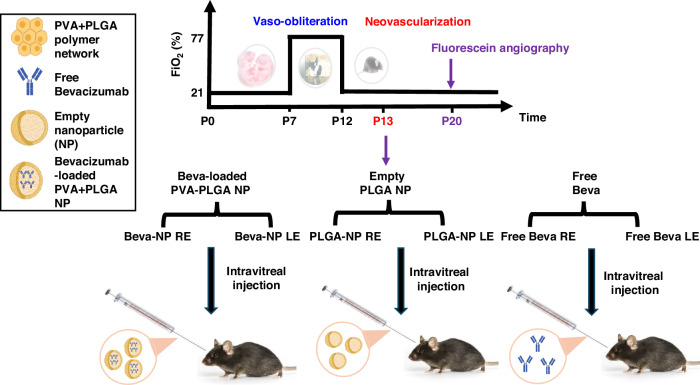
In vivo mouse model of oxygen-induced retinopathy and intravitreal bevacizumab administration. Newborn mice and their nursing dam are exposed to 77 ± 2% oxygen from postnatal day (P)7 to P12, before returning to room air. Hyperoxia from P7 to P12 simulates Vaso-obliteration in the retina of the mice, while moving the mice to room air or relative hypoxia from P12 onwards simulates Neovascularization, similar to seen in retinopathy of prematurity. At P13, intravitreal injection is administered to right eyes only. Left eyes serve as control. Live FA occurs at P20 following anesthesia. PLGA (poly-d,l-lactide-co-glycolic acid); Beva denotes Bevacizumab, Control denotes untreated left eye, RE denotes right eye, LE denotes left eye, PLGA-NP-RE refers to the right eye treated with empty PLGA-PVA nanoparticles, while PLGA-NP-LE signifies untreated contralateral left eye of the same mouse. Beva-NP-RE refers to the right eye of a mouse treated with Bevacizumab-loaded PLGA-PVA nanoparticles, while Beva-NP-LE signifies untreated contralateral left eye of the same mouse. Free Beva denotes free conventional unencapsulated Bevacizumab. Free Beva RE denotes right eye treated with Free, unencapsulated Bevacizumab, while Free Beva LE denotes contralateral untreated left eye.

Newborn C57BL6/J mice were exposed to 77 ± 2% oxygen in a hyperoxia chamber for 5 days to induce oxygen-induced retinopathy (OIR)from postnatal day (P) 7 to P12, before returning them to room air. OIR exposure proceeded according to an in vivo mouse model of OIR by Mezu-Ndubuisi,^1^ specific for in vivo FA, which was modified from the mouse OIR model for retinal flat mounts by Dr. Lois Smith.^29^ The in vivo OIR model differs from the traditional OIR model in the higher level of oxygen needed to simulate visible retinal artery tortuosity and retinal vein dilation, as well as a limit of 6 mice per cage in hyperoxia to avoid exaggerated vasculopathy from growth restricted mice.^30–32^ Vanhaesebrouck et al noted that mice from larger litters had lower body weight, lower circulating levels of insulin growth factor and more severe OIR, while Stahl et al showed that pups with poor postnatal weight gain had delayed and markedly prolonged vaso-obliteration compared to larger body weight pups.^30,31^ Newborn litters, in our study, were reduced to six pups at P5, prior to hyperoxia exposure, to avoid growth-restriction from nursing large litters, which worsens the OIR phenotype observed during in vivo imaging. The nursing dams were rotated between OIR and RA every 24 to 48 h to avoid the toxic effects of oxygen toxicity on adult mice. Mice remained with nursing dam in room air until intravitreal injection of experimental drug at P13 and in vivo FA at P20 (Fig. 1).

### Nanoparticle fabrication

#### PLGA nanoparticle synthesis

**Fig. 2:**
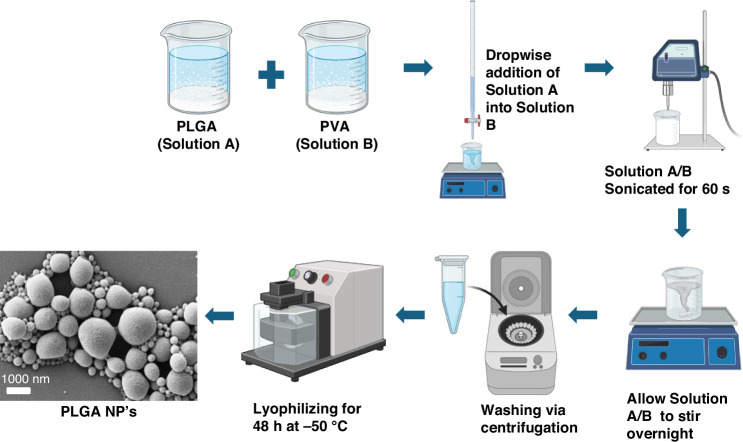
Schematic of fabrication of PLGA nanoparticle. Figure 2 shows the fabrication process used for the synthesis of PLGA nanoparticles. 4% PVA solution and DCM solvent were used in Experiment 1, and optimized to 2% PVA and ethyl acetate solvent in Experiment 2. A similar approach is used for the Bevacizumab loaded nanoparticles, where Bevacizumab is added to PLGA solution. For the Bevacizumab-loaded PLGA nanoparticle fabrication, a drug: polymer ratio of 1:100 was used in Experiment 1, while an optimized drug: polymer ratio of 1:10 was used in Experiment 2. PLGA denotes (poly-d,l-lactide-co-glycolic acid); PVA denotes poly vinyl alcohol; NP denotes nanoparticle; DCM denotes Dichloromethane.

Two experiments were conducted to synthesize PLGA nanoparticles using the double emulsion-solvent evaporation method,^33^ with the aim of optimizing the size and drug dissolution profile of the nanoparticles. In Experiment 1, 4% Polyvinyl alcohol (PVA, 99.9% hydrolyzed, 10.000 MW) solution was prepared by adding 1 g of PVA powder to 25 ml of water, and continuously stirred at 500 rpm for 30 minutes at 60 °C. For PLGA nanoparticle synthesis, 0.1 g of PLGA (50:50, acid-terminated, inherent viscosity 0.15 g/dL, molecular weight (MW): 15,000 g/mol, Polysciences, Inc., Warrington, PA) was added to 4 mL Dichloromethane (DCM) to make Solution A. Solution A was vortexed for 5 s to prevent PLGA adherence to the walls, followed by sonication at 60% amplitude for 60 s to form a uniform PLGA solution (Fig. 2). Solution A was added dropwise to 20 mL of 4% aqueous PVA solution constantly stirring at 600 rpm. The resultant Solution B was sonicated at 60% amplitude for 60 s and stirred overnight before centrifuging for 15 min at 10,000 g and washing with ultrapure distilled water three times to remove impurities. The final white pellet of nanoparticles were lyophilized at −50 °C for 3 days prior to characterization. In Experiment 2, the concentration of the PVA solution was reduced from 4% to 2%, and the solvent was changed from DCM to ethyl acetate, while all other steps in Experiment 1 remained constant. Figure 1 shows the schematic of nanoparticle fabrication.

### Bevacizumab-loaded PLGA nanoparticle synthesis

Two experiments were conducted to optimize encapsulation of Bevacizumab in PVA-PLGA copolymer nanoparticles. Using the single emulsion solvent evaporation method, as previously described,^24^ water in oil in water (w/o/w) nanoparticles was fabricated. In Experiment 1, 1 mg Bevacizumab (MedChem Express) was added to 100 mg PLGA in 1 ml DCM to obtain a drug; polymer ratio of 1:100. The subsequent solution was added to 4 ml of 2% PVA on a magnetic stirrer at 400 rpm and sonicated at 60% amplitude for 60 s with 10 s pulse time. The subsequent solution was added dropwise using a 5 ml syringe and 21 G needle into 7.5 ml of 2% PVA on a magnetic stirrer at 400 rpm. It was constantly stirred overnight to evaporate the organic solvent. The solution was then filtered with a 0.2 µm syringe filter and washed three times with distilled water after 15,000 rpm centrifugation for 15 minutes. The nanoparticles were then frozen at −20 °C and then lyophilized for 48 h at 50 °C. In Experiment 2, we varied the drug: polymer ratio to 1:10, and the solvent used to dissolve Bevacizumab and PLGA was changed from DCM to ethyl acetate, while keeping all other parameters constant. This is depicted in the graph of Bevacizumab-loaded nanoparticle drug release profile (Fig. 2).

### Intravitreal injection

Following characterization, only the optimized nanoparticles from Experiment 2 were utilized to demonstrate efficacy in OIR. At P13, optimized Bevacizumab-loaded nanoparticles and Empty PLGA nanoparticles from Experiment 2, and Free Bevacizumab were prepared for intravitreal injection. In the cage of 6 mice that had been exposed to hyperoxia, 3 received empty PLGA nanoparticles and 3 received Bevacizumab-loaded PLGA nanoparticles intravitreally injected to their right eye (RE). The left eye (LE) of each mouse that was exposed to hyperoxia did not receive any injection. In brief, mice were anesthetized with an intraperitoneal (IP) injection of ketamine (100 mg/kg) and xylazine (10 mg/kg) and placed on a heated pad at 37 °C. The right eye was visualized under a dissecting microscope, and the skin around the eye was cleansed with ethanol. After a 1 mm aperture, posterior to the superotemporal limbus, was made with a 30-gauge needle lancet, a 33-gauge Hamilton needle was inserted and directed towards the vitreous, avoiding the lens. 1.25 µg/0.5 µl of 25 mg/ml Bevacizumab-loaded PLGA or equivalent concentration of Empty PLGA nanoparticles was given over 30 s to enable proper distribution. Ofloxacin ophthalmic solution (AdvaCare Pharma, Cheyenne, WY) was applied to eyes twice daily for 4 days.

### Fluorescein angiography

At P20, following anesthesia with ketamine and xylazine, IP injection of 10% fluorescein sodium (100 mg/kg) (AK-FLUOR; Akorn, Decatur, IL) was administered, prior to placing a mouse on a Micron IV small animal ophthalmic imaging system (Phoenix Research) for FA. Photos of 50 degree views of the retina were obtained from right eyes of mice injected with Bevacizumab-loaded PLGA nanoparticles (Beva-NP-RE) as well as the contralateral untreated left eyes of the same mouse (Beva-NP-LE). Similarly, FA was obtained from the right eyes of the Empty PLGA nanoparticle treated eyes (PLGA-NP-RE) followed by the contralateral untreated left eyes (PLGA-NP-LE). Over 50 to 75 retinal photos were obtained per mouse capturing successive views and quadrants, depending on the clarity of the optical media and tolerance of the animal.

#### Quantification of retinal vascular parameters

Using a customized MATLAB™ program, retinal artery tortuosity (RAT), retinal vein width (RVW), and retinal avascular area (RAA) was quantified as described by Mezu-Ndubuisi,^1^ and freely available at www.QuantBV.com. In brief, for image analysis, one image from each mouse with the optic nerve centrally located (central image) is used to determine RAA, to ensure evaluation of a uniform area in each mouse with the automated RAA determination, while one image with a peripherally located optic nerve (peripheral image) is selected, to ensure adequate length of retinal artery and vein for semi-automated calculation of RAT and RVW respectively.

To measure the Retinal Vascular Area, the central image was uploaded into QuantBV, which automatically converts it into a black and white binary image, and then a grayscale image to automatically calculate the total area in the image occupied by all retinal vessels and capillaries, expressing it as a percentage of the total retinal area analyzed. For RVW and RAT, the same peripheral image is used. The widest vessel is selected as the vein, and the most tortuous vessel is selected as the artery. In room air mice, vessels are not dilated or tortuous, but the veins are larger than the arteries, and the arteries are noted to have successive dichotomous branching or bifurcation from the optic nerve. For Retinal Vein Width, a peripheral image was uploaded into the program, and using a semi-automated method we manually traced the width of the retinal vein, starting from the edge of the optic nerve, and traveling along the length of the vessel to a pre-determined and validated distance of 275 ± 25 μm from the optic nerve. The program then automatically calculates and average of all width points selected. To determine Retinal Arterial Tortuosity, we manually selected equidistant points along the length of the artery, from the base of the optic nerve outwards up to a branch point, up to a pre-determined and validated distance of 175 ± 25μm for RA mice and 275 ± 25 μm for OIR mice. Room air arteries are non-tortuous with only physiologic dichotomous branching, and so the measurement ends at the first branching for RA mice to avoid over-estimating the RAT. The software then automatically calculates the total length traced, and projects a straight line connecting the first and last selected points. The tortuosity index was calculated as the ratio of the actual length of the artery traced to the projected length of the straight line. A score of 1 indicated a perfectly straight vessel, while values greater than 1 represent increasing levels of tortuosity.

For each experimental group, newborn mice from the same litter were exposed to OIR and not mixed with different litters, while mice from different litters were used for retinal image analysis. However, some mice were lost to complications of intravitreal injection and cataracts during FA limiting image quality. Therefore, retinal images derived from mice born in separate litters were used for retinal image analysis.

### Characterization of nanoparticles

#### Scanning electron microscopy (SEM)

SEM is employed to observe and the surface morphology and nanoparticle size. Small aliquots of the nanoparticle suspensions were allowed to dry onto carbon sticky tapes mounted on aluminum stubs. The samples were sputter coated with gold metal prior to loading into a Zeiss Auriga field emission Scanning Electron microscope for morphologic analysis and image capture using 5 kV and a 3.5 mm average working distance. The high resolution SEM images were selected to determine average nanoparticle size and distribution by using ImageJ and data processing with Origin. A semi-automated approach was used to enable accuracy and rapid size analysis.

### Transmission electron microscope

The drug-loaded PLGA nanoparticles were analyzed using a Hitachi 7650 transmission electron microscope (TEM) to visualize individual nanoparticles, examine the internal nanostructure and indicate the presence and integrity of both empty and drug-loaded PLGA nanoparticle. TEM verified the encapsulation of Bevacizumab within the PLGA matrix, by revealing a defined core structure within each particle.

### Fourier transform infrared spectroscopy (FTIR)

FTIR spectroscopy is a highly sensitive, non-destructive technique for evaluating protein secondary structure within nanoparticles.^20^ FTIR is utilized to identify molecular structures based on their infrared (IR) absorption features, which correlate with molecular vibrations. IR absorption spectra were collected for the Empty PLGA and Bevacizumab-loaded nanoparticles. The spectra were recorded using a Perkin Elmer Spectrum 100 Fourier Transform Infrared Spectroscopy Spectrometer (PerkinElmer, Downers Grove, IL) equipped with a Specac Golden Gate single-reflection attenuated total reflectance (ATR) accessory. A total of 64 scans were averaged with a resolution of 4 cm^−1^ over a region of 500–4000 cm^−1^. FTIR is a type of spectroscopy that detects changes in functional groups that compose biomolecules through the vibration and rotation of molecules due to infrared radiation at a specific wavelength.^34^ This identifies structural differences in the molecular binding between entities, thereby confirming the presence of the drug in the nanoparticle polymers.

### In vitro drug release study

In vitro drug release profile of Bevacizumab from the prepared nanoparticles was evaluated by quantifying the cumulative amount of Bevacizumab in a 500 µl phosphate buffer with pH 7.4 over time. 50 µl of the supernatant was collected at 0, 6,12, 24, 48, 72, 96, 120 h time points by ultracentrifugation in a Benchmark M-12 ultra-centrifuge (Benchmark Scientific) at 16,000 g for 15 min. Bicinchoninic acid assay (BCA) analysis was performed to quantify the protein release. Briefly, 20 µL of the supernatant was added to 180 µL solution of BCA reagent A and B and incubated at 37 °C for 30 min. It was then analyzed in a nanodrop (Thermoscientific), at each time point.

### Drug loading and encapsulation efficiency

To estimate drug loading, Bevacizumab-loaded nanoparticles were added to 500 µL of dimethyl sulfoxide (DMSO) to disintegrate the PLGA polymers and release the Bevacizumab protein. The supernatant was isolated by ultracentrifugation in a Benchmark M-12 ultra-centrifuge (New Jersey) at 16,000 g for 30 min. BCA analysis was performed to quantify the protein released. The Drug Loading Capacity (DL) % and Encapsulation Efficiency (EE) % were determined indirectly. %DL= [(total amount of drug – amount of supernatant) ÷ nanoparticle mass] ×100. %EE = [(total amount of drug – amount of drug supernatant) ÷ total amount of drug] ×100.

### Statistical methods

Data is presented as mean ± standard deviation (SD). Statistical analyses were performed using Graph Pad Prism software V10.4.1. Unpaired Student’s *t*-test was calculated. *P* ≤  0.05 was considered significant.

## Results

### Scanning electron microscopy (SEM)

**Fig. 3:**
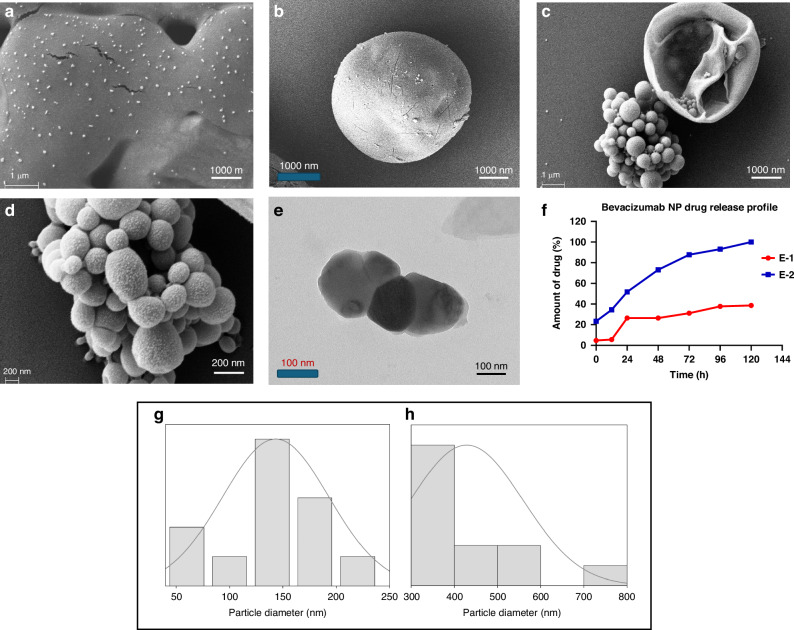
SEM, TEM, and Drug release profile of Bevacizumab nanoparticle. **a** SEM image of Bevacizumab-loaded PVA-PLGA NPs from Experiment 1 with a magnification of 1000 nm. **b** Single image of a Bevacizumab-loaded PVA-PLGA nanoparticle from Experiment 2 with a magnification of 1000 nm. **c** SEM of Empty PVA-PLGA nanoparticle with a burst release from Experiment 1 with a magnification of 1000 nm. **d** SEM image of Empty PLGA nanoparticles from Experiment 2 with a magnification of 200 nm. **e** TEM image of Bevacizumab-loaded PVA-PLGA nanoparticles showing the shape of nanoparticles and drug contained within the nanocapsule at a magnification of 1000 nm. **f** Drug release profile for Experiment 1, showing 26.4% release in 24 h and 31.1% at 72 h. In Experiment 2, 51.7% was released in 24 h and 80 at 72 h. **g** The average size of the Bevacizumab nanoparticles ranged from 50 nm to 250 nm, with an average of 143 ± 49 nm. **h** The size of PLGA nanoparticles ranged from 300 nm to 800 nm, with an average of 428 ± 131 nm. SEM denotes Scanning Electron Microscopy; TEM denotes Transmission Electron Microscopy, NP denotes nanoparticle, PLGA denotes PLGA denotes (poly-D,L-lactide-co-glycolic acid), PVA denotes polyvinyl alcohol, E1 denotes Experiment 1, E2 denotes Experiment 2, Empty PLGA denotes PLGA nanoparticle without drug incorporated. Beva PLGA denotes Bevacizumab nanoparticles fabricated in Experiment 2 using 2% PVA and ethyl acetate solvent.

The Empty and Bevacizumab-loaded PLGA nanoparticles were characterized using SEM to reveal a spherical morphology. SEM images from Experiment 1with 4% PVA, drug; polymer ratio of 1:100, and DCM solvent were non-homogenous with ill-defined with sub-optimally shaped nanospheres, suggestive of the presence undissolved polymers and some crystallization due to higher pH solvent, as seen in Bevacizumab NP (Fig. 3a) and Empty PLGA nanoparticles (Fig. 3c). SEM images from Experiment 2 with 2% PVA, a drug: polymer ratio of 1:10 and ethyl acetate solvent had a more distinct spherical structure and morphology with a reduced burst effect, resulting in more optimized nanoparticles, as seen in Bevacizumab nanoparticles (Fig. 3b) and Empty PLGA nanoparticles (Fig. 3d). The average size of the Bevacizumab nanoparticles ranged from 50 nm to 250 nm, with an average of 143 ± 49 nm (Fig. 3g). The size of PLGA nanoparticles ranged from 300 nm to 800 nm, with an average of 428 ± 131 nm (Fig. 3h).

### Transmission electron microscopy (TEM)

The drug loaded PLGA nanoparticles were subjected to TEM (Hitachi 7650 TEM) to analyze their structure, and confirm the presence of drug enclosed within the capsule of the PLGA polymer. TEM of Bevacizumab nanoparticles from Experiment 2 more distinctly showed the presence of Bevacizumab inside the nanoparticle capsule compared to Experiment 1, (Fig. 3e).

### Bevacizumab drug release profile

Bevacizumab-loaded PLGA nanoparticles showed a biphasic in vitro drug release profile. In Experiment 1, there was a 26.4% release in 24 h with a peak release of 31.1% at 72 h. In Experiment 2, 51.7%, was released in 24 h, and increased to 80% at 72 h (Fig. 3f).

### Fourier transform infrared spectroscopy (FTIR)

**Fig. 4:**
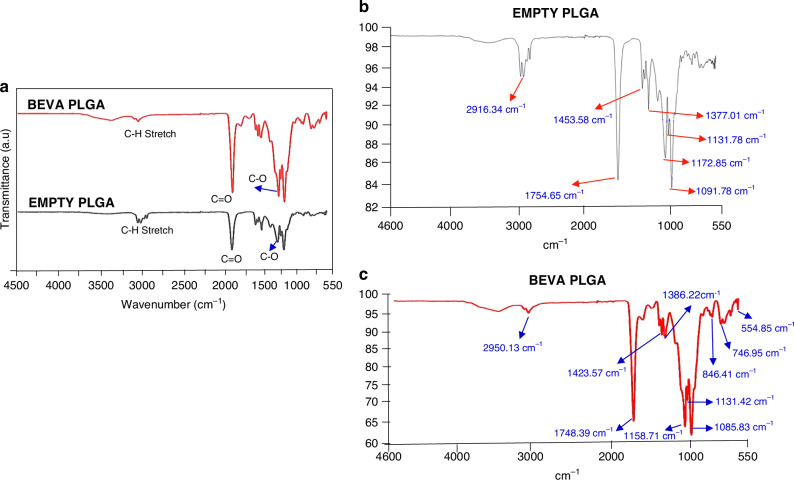
FTIR of Bevacizumab-loaded and Empty PLGA Nanoparticle. FTIR identifies functional C,H,O groups on the Bevacizumab-loaded PLGA nanoparticles and Empty PLGA nanoparticles based on their infrared absorption spectra peaks in the graph. Bevacizumab nanoparticles show comparable peaks to PLGA spectra and additional unique wavelengths. **a** Merged Bevacizumab and PLGA FTIR. **b** Bevacizumab FTIR. **c** PLGA FTIR. FTIR denotes Fourier Transform Infrared Spectroscopy; PLGA denotes poly lactic – co glycolic acid; Beva denotes Bevacizumab; C denotes Carbon; H denotes Hydrogen, O denotes Oxygen. Empty PLGA signifies PLGA nanoparticle with no drug incorporated. Beva PLGA signifies PLGA nanoparticle with Bevacizumab incorporated within.

FTIR was performed to determine the structural changes of the Bevacizumab within the PLGA nanoparticles, and identify the functional groups and polymer backbone present. The FTIR spectra of empty and drug-loaded nanoparticles shows characteristic peaks or absorption bands (Fig. 4e). The FTIR spectra of the empty nanoparticles exhibited characteristic peaks at 1754 cm⁻¹, corresponding to the carbonyl group (C=O), a C-H stretch at ~2916 cm⁻¹, and a C-O-C stretch at 1131 cm⁻¹, attributed to PLGA. The Bevacizumab-loaded PLGA nanoparticles IR spectra showed similar peaks to empty PLGA, with additional peaks with lower intensity observed, such as at 1645 cm⁻¹ and 1531 cm⁻¹, which could be due to C-O and C-H vibrations from the antibody.

### Bevacizumab drug loading and encapsulation efficiency

In Experiment 1, the Bevacizumab % DL was 1.4% and %EE was 78%. In Experiment 2, the % DL was 2.3% and %EE was at 80%.

### Intravitreal Bevacizumab nanoparticles ameliorated retinal vasculopathy

**Fig. 5:**
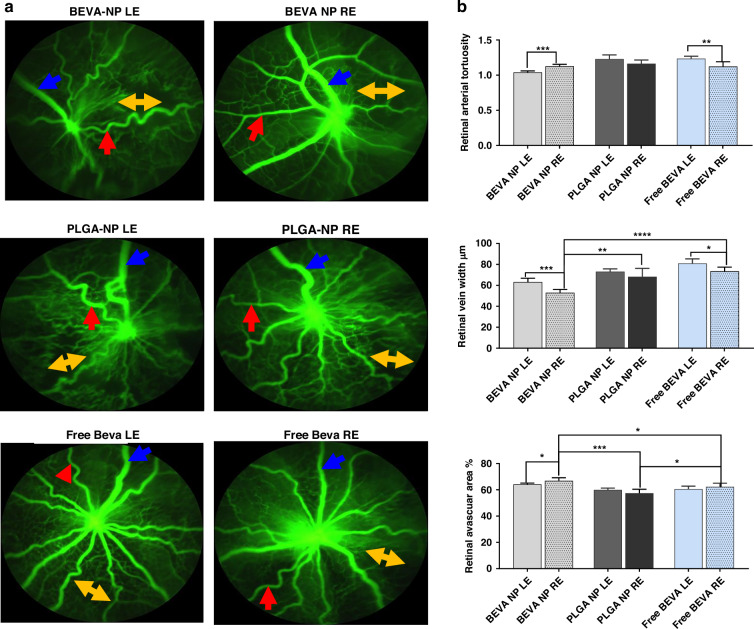
Fluorescein Angiograms of P20 OIR mice treated with Bevacizumab-loaded or Empty PLGA Nanoparticles. Representative Fluorescein Angiogram images at post-natal day 20 are shown in **a**. The Bevacizumab-treated right eye show increased avascularity as well as reduced vein tortuosity and reduced arterial tortuosity compared to mice with PLGA-RE. The untreated (control) left eye shows greater vein tortuosity and higher retinal artery tortuosity. Treatment with empty nanoparticles in the right eye shows similar results to the untreated left eye, with greater vein tortuosity and less total avascular capillary areas. **b** shows the quantification of the retinal vascular parameters. Retinal Avascular Area was increased in Beva-NP-RE compared to PLGA LE, while Retinal artery tortuosity and retinal vein width were decreased in Beva-NP-RE compared to PLGA RE. The untreated Beva-NP-LE and PLGA-NP-LE were similar in their retinal avascular areas and tortuosity to PLGA LE, but the retinal vein width was reduced in Beva-NP-LE, reflecting the variability in the morphology and function of retinal veins or possible contralateral absorption from the treated Beva-NP-RE. OIR denotes oxygen-induced retinopathy; RAA denotes Retinal Avascular Area, RAT denotes Retinal Artery Tortuosity, RVW denotes Retinal Vein Width, P denotes postnatal day; PLGA (poly-d,l-lactide-co-glycolic acid); Beva denotes Bevacizumab, Control denotes untreated left eye, RE denotes right eye, LE denotes left eye, PLGA-NP-RE refers to the right eye treated with empty PLGA-PVA nanoparticles, while PLGA-NP-LE signifies untreated contralateral left eye of the same mouse. Beva-NP-RE refers to the right eye of a mouse treated with Bevacizumab-loaded PLGA-PVA nanoparticles, while Beva-NP-LE signifies untreated contralateral left eye of the same mouse. Free Beva denotes free conventional unencapsulated Bevacizumab. Free Beva RE denotes right eye treated with Free Bevacizumab, while Free Beva LE denotes contralateral untreated left eye. Red arrow shows arteries; Blue arrow shows veins; orange double arrow shows retinal capillary areas; *P < 0.05, **P < 0.01, ***P < 0.001, ****P < 0.0001.

To determine the efficacy of the optimized bevacizumab nanoparticles in ameliorating retinal vasculopathy, we injected the right eyes only of mice exposed to OIR with either Empty PLGA nanoparticles (PLGA-NP RE), Bevacizumab-loaded nanoparticles (Beva-NP RE), or free, unencapsulated Bevacizumab protein (Free Beva RE). The contralateral left eyes of the same mice exposed to OIR did not received any Empty PLGA nanoparticles (PLGA-NP LE), Bevacizumab-loaded nanoparticles (Beva-NP LE), or free, unencapsulated Bevacizumab protein (Free Beva LE). We performed fluorescein angiography in live anesthetized mice (Fig. 5a). Following FA, we quantified retinal vascular parameters using QuantBV, our customized and validated MATLAB program, (Fig. 5b) (Table 1). Beva-NP RE mice had more avascular retinal areas than PLGA-NP RE mice (*P* = 0.0001), confirming Bevacizumab’s anti-angiogenic effect. Beva-NP RE had more retinal avascularity than the contralateral Beva-NP LE of the same mice (*P *= 0.0224) (Table 1). This indicated that nanoparticles did not penetrate the contralateral left eyes and their effect was limited to the injected right eyes. The Beva-NP LE showed more retinal avascularity than PLGA-NP-LE, likely indicating regional differences in the central FA images used for the analysis, (*P* = 0.0001). PLGA-NP RE showed similar retinal avascular areas than PLGA-NP LE (*P* = 0.0630). For the Bevacizumab nanoparticle treated mice, the retinal artery tortuosity index was decreased in the Beva-NP RE compared to the contralateral untreated Beva-NP LE (*P* = 0.0001). Beva-NP RE mice had increased retinal avascular areas compared to Free Beva RE mice (*P* = 0.0112), confirming increased bioavailability of Bevacizumab nanoparticles over free drug, leading to enhance antiangiogenic effect (Table 1). The retinal arteries were less tortuous in Beva-NP RE of mice than the PLGA-NP RE (*P* < 0.0001). As expected, there was no difference in the retinal artery tortuosity in the right eyes and left eyes of mice treated with Empty PLGA nanoparticles. The retinal arteries of Free Beva-RE mice were more tortuous than Beva-NP RE mice (*P* = 0.023) confirming the superior efficacy of nanoparticle versus free Bevacizumab. We compared retinal vein widths, and noted that the retinal veins were less dilated in Beva-NP RE compared to Beva-NP LE (*P* = 0.0004) and PLGA-NP RE (*P* = 0.0014) (Table 1). PLGA-NP RE and PLGA-NP LE had similar retinal vein width (*P* = 0.1802). However, Beva-NP LE had reduced retinal vein width than PLGA-NP LE, reflective of the variability of retinal vein width in OIR mice. Free Beva NP mice has increased retinal vein width than Beva-NP RE mice (*P* < 0.0001), further confirming increased retinal efficacy of nanoparticle formulation of Bevacizumab over free drug.

**Table 1:** Retinal Vascular Parameters in Free Bevacizumab, Bevacizumab-loaded and Empty nanoparticle treated OIR mice

Parameter	Beva-NP LE		Beva RE	PLGA-NP LE	PLGA RE	Free Beva LE	Free Beva RE
	*n*	6	6	6	6	6	6
RAA(%)	**Mean**	64.07	66.86	59.90	57.46	60.58	62.28
	**SD**	1.020	2.323	1.370	2.947	2.174	2.770
P-value	Beva-NP RE vs Beva-NP LE		0.0224				
	Beva-NP RE vs PLGA-NP RE		0.0001				
	PLGA-NP RE and PLGA-NP LE		0.0959				
	Beva-NP LE vs PLGA-NP LE		0.0001				
	Free Beva RE vs Beva-NP RE		0.0112				
	Free Beva LE vs Beva-NP LE		0.0052				
	Free Beva RE vs Free Beva LE		0.2639				
RAT	**Mean**	1.126	1.039	1.163	1.230	1.235	1.124
	**SD**	0.028	0.021	0.0526	0.058	0.035	0.067
P-value	Beva-NP RE vs Beva-NP LE		0.0001				
	Beva-NP RE vs PLGA-NP RE		<0.0001				
	PLGA-NP RE and PLGA-NP LE		0.0630				
	Beva-NP LE vs PLGA-NP LE		0.1651				
	Free Beva RE vs Beva-NP RE		0.0152				
	Free Beva LE vs Beva-NP LE		0.0001				
	Free Beva RE vs Free Beva LE		0.0049				
RVW (µm)	**Mean**	63.21	52.96	73.16	68.23	80.95	73.47
	**SD**	3.585	3.130	2.537	7.978	4.339	3.933
P-value	Beva-NP RE vs Beva-NP LE		0.0004				
	Beva-NP RE vs PLGA-NP RE		0.0014				
	PLGA-NP RE and PLGA-NP LE		0.1802				
	Beva-NP LE vs PLGA-NP LE		0.0002				
	Free Beva RE vs Beva-NP RE		0.0001				
	Free Beva LE vs Beva-NP LE		<0.0001				
	Free Beva RE vs Free Beva LE		0.0107				

Table 1 shows the measured values for retinal vascular parameters obtained from fluorescein angiograms in mice with oxygen-induced retinopathy following treatment with intravitreal Bevacizumab-loaded PLGA nanoparticles or Empty PLGA nanoparticles to the right eyes only. The left eyes were untreated and served as controls.

PLGA poly-D,L-lactide-co-glycolic acid, Beva denotes Bevacizumab, LE denotes untreated left eye, RE denotes treated right eye, PLGA-NP RE refers to the right eye treated with empty PLGA-PVA nanoparticles, while PLGA-NP LE signifies untreated contralateral left eye of the same mouse. Beva-NP RE refers to the right eye of a mouse treated with Bevacizumab-loaded PLGA-PVA nanoparticles, while Beva-NP LE signifies untreated contralateral left eye of the same mouse. Free Beva denotes free conventional unencapsulated Bevacizumab. Free Beva RE denotes right eye treated with Free Bevacizumab, while Free Beva LE denotes contralateral untreated left eye. *P* < 0.05 is statistically significant.

## Discussion

We present the first PVA-PLGA nanoparticle incorporated with Bevacizumab in ethyl acetate solvent for intravitreal administration in an in vivo mouse model of OIR. Clinical use of Bevacizumab and other anti-VEGF therapies for drug treatments for ROP are complicated by their systemic absorption and repeat dosing, due to a short half-life, leading to systemic toxicity to developing organs.^15,18,35^ We optimized the fabrication of intravitreal Bevacizumab-loaded nanoparticles, which demonstrated improved retinal vasculopathy with FA in mice exposed to OIR.

Our SEM images confirmed the spherical shape of both Empty and Bevacizumab-loaded PLGA nanoparticles (Fig. 3). In Experiment 1, using a higher concentration of PVA (4%) and DCM as the solvent, we produced nanoparticles with less-defined surfaces and visible signs of crystallization due to undissolved polymers and phase separation. This is consistent with reports that higher PVA concentrations can lead to non-uniform particles due to slower dissolution rates and more challenging emulsion stabilization.^36^ Using 2% PVA and ethyl acetate as a solvent in Experiment 2 produced more uniform and spherical particles with more distinct morphology. Ethyl acetate, with its lower pH, likely stabilized the PVA within the PLGA matrix, enhancing nanoparticle formation and drug release. We achieved polymer sizes in the nano scale as low as 50 to 200 nm. Polymer size of about 200 nm result in retinal permeation, while larger nanoparticle polymers of ~1000 nm or microspheres have been localized in the vitreous.^37^

Our studies determined percent drug loading, a representation of the amount of drug bound to the nanoparticle and their interaction, which depends on the chemical structure of the drug and polymer and drug loading conditions.^38^ We achieved a drug loading of 1.4% capacity and encapsulation efficiency of 78% for Experiment 1, and a 2.3% drug loading capacity and encapsulation efficiency of 80%, in Experiment 2, which are in agreement with existing literature,^20,39^ indicating effective encapsulation of Bevacizumab by PVA-PLGA copolymer. A high encapsulation efficiency suggests retention of a significant amount of Bevacizumab within the nanoparticles, minimizing drug wastage. An EE% of 78-80% observed in our study is higher than that reported for other Bevacizumab nanoparticle studies, including 47.625 ± 7.022% for Bevacizumab encapsulated in nanoliposomes for human umbilical vein endothelial cells (HUVEC),^40^ 32.1  ±  6.6% using Bevacizumab encapsulated in echogenic liposomes for HUVECs.^41^ Our higher EE% for Bevacizumab is similar to that achieved by Sousa et al.^20^ and Fatima et al of about 82%.^21^

Controlled and sustained release of Bevacizumab nanoparticles is critical to its efficacy. Bevacizumab nanoparticle release is influenced by several factors, including polymer degradation, protein diffusion, molecular size, isoelectric point, nanoparticle porosity. The drug release process has an initial “burst” release as the PLGA matrix erodes, quickly releasing Bevacizumab protein located near the nanoparticle surface, followed by a sustained release.^37,42^ In this study, optimized Bevacizumab nanoparticles with PVA-PLGA co-polymer had a burst release of 51.7%, and was released in 24 h, followed by 80% at 72 h. This compares favorably to Liu et al.'s study of Bevacizumab-loaded nanoparticles with 30% release in 24 h, followed by 65% release by 72 h.^43^ Sousa et al. reported a slower release pattern of 18% released at 24 h and 50% by 72 h for Bevacizumab encapsulated in PLGA nanoparticles with a molecular weight of 0.45 g/dl,^20^ while our study used a PLGA molecular weight of 0.20 g/dl in a PVA copolymer with increased release efficiency. Varshochian et al. showed 20% release of Bevacizumab from PLGA nanoparticles within the first 24 h, and 65% by 72 h,^44^ a delayed release likely due to differences in polymer molecular weight and surfactant choice.

Changing the solvent from DCM to ethyl acetate in our study optimized Bevacizumab’s encapsulation and enhanced its electrostatic interactions enabling efficient release. Polymer degradation, influenced by hydrolysis, enzymatic cleavage, and polymer characteristics, drives release as Bevacizumab’s large size slows diffusion.^20^ Ethyl acetate optimized Bevacizumab’s encapsulation and enhanced its electrostatic interactions, enabling efficient release.. The solvent change enhanced Bevacizumab’s polarity, improving hydrophobic interactions and facilitating release.^20,45^ Ethyl acetate’s higher boiling point (77 °C versus 39.6 °C for DCM) better controlled evaporation, optimizing protein encapsulation.^46^

Our study showed that Bevacizumab nanoparticles administration was more effective than Free Bevacizumab, consistent with other literature. Zhang et al demonstrated controlled release of 40% of Bevacizumab from PLGA nanoparticles in 7 days compared to mostly all of the free Bevacizumab was released in the first 2 to 7 hours, resulting in enhanced inhibition of angiogenesis in the retina in OIR mice compared to free Bevacizumab.^47^ Bevacizumab-PLGA microspheres treatment in the left eyes of healthy rabbits were found to have achieved a higher concentration and longer half-life (10 days versus 4 days) than conventional formulation of soluble Bevacizumab in solution following treatment in the contralateral right eyes.^48^ Bevacizumab’s half-life in adults with choroidal neovascularization was 6.7 days.^49^ Bevacizumab administered intravitreally in adults with age-related macular degeneration necessitates frequent injections to sustain therapeutic levels, with increased risk of adverse effects from the intravitreal injection technique, such as vitreous hemorrhage, retinal detachment, endophthalmitis, and cataracts.^50,51^ Systemic exposure to free antibody can produce serious adverse events such as gastrointestinal perforation, high grade thromboembolic events, wound dehiscence, bleeding, hypertension, and proteinuria,^52–54^ while encapsulation of Bevacizumab within PLGA nanoparticles offers a controlled-release profile that enhances drug pharmacokinetics, protects the antibody from rapid enzymatic degradation and improves efficacy, prolongs intraocular retention, reduces the frequency of administrations, and minimizes peak systemic concentrations.^55,56^

Bevacizumab nanoparticles improved the abnormal retinal vascular features in OIR mice, akin to the observed improved diabetic macular edema and best corrected visual acuity seen following unilateral intravitreal treatment of Bevacizumab nanoparticle in adult patients.^57^ The retinal avascular area in our study was increased in Beva-NP RE compared to the untreated left eyes of Beva-NP LE mice. The Beva-NP RE had increased retinal avascularity than the PLGA-NP RE that received empty nanoparticle injections. Increase in retinal avascularity would be the expected pharmacodynamic effect of Bevacizumab treatment due to its antiangiogenic properties. The retinal arteries were less tortuous and the retinal veins were less dilated in Bevacizumab treated right eyes compared to contralateral untreated left eyes and empty PLGA treated mice, similar to the findings with our prior use of VEGFA_165a_ microparticles.^23^ Retinal artery tortuosity and retinal vein dilation indicate OIR severity,^1,3^ and signify plus disease, a marker of severe ROP in infants.^16,58^ It is worthy of note that anti-angiogenic Bevacizumab and pro-angiogenic VEGFA_165a_ have opposing effects on retinal vascularization, and that Bevacizumab nanoparticles were much smaller than microparticles. There appeared to be some drug effect on the untreated contralateral Beva-NP LE, likely due to minimal effect of the lactic acid in PLGA. Although the retinal veins were less dilated in Beva-NP RE than the contralateral Beva-NP LE, the Beva-NP LE had smaller retinal veins than the PLGA-NP LE. The retinal veins have more variable widths in response to hyperoxia exposure, and therefore showed mouse to mouse variability in horizontal vein width measurements. As we published previously, arterial tortuosity is a more reliable marker of OIR severity and recovery than retinal vein dilation or avascularity.^1^ This contralateral drug effect of reduced retinal vein width in the Empty PLGA nanoparticle treated right eyes was also observed in our prior use of microparticles,^23^ and could be due to the smaller nanoparticle size which improved drug penetration and bioavailability, as well as the effect of the lactate released from the nanoparticles. Encapsulated PLGA degrades intraocularly to release free lactate, which has been observed to induce tissue vascular regeneration during wound healing through hypoxia-inducible factor-activation, release of macrophage VEGF, and stimulation of pro-angiogenic growth factor release from endothelial cells.^59,60^ Further investigation of the selective effects of PLGA on retinal veins would shed more insights into its nanoparticle retinal effects, but was beyond the scope of our current study. Our results are promising for potential clinical translation of intravitreal Bevacizumab-loaded PLGA nanoparticle optimized with PVA and ethyl acetate to reduce pathologic retinal neovascularization, retinal artery tortuosity and retinal vein dilation, features of plus disease or severe ROP, in premature infants with retinopathy of prematurity.

Limitations of our study include complications of intravitreal injection causing cataracts and vitreous hemorrhage, limiting the quality of some FA images. Poor quality images were excluded from analysis. Two to three litters per cage in each experimental group were studied to ensure adequate numbers for mice for retinal image analysis. Eight cages (*n* = 6) total were used for the experiment, with a potential of 48 mice (3 cages for PLGA-NP, 3 cages for Beva-NP, and 2 cages for Free Beva). Only 24 underwent in vivo FA. 24 mice were excluded from in vivo FA due to demise from maternal infanticide after intravitreal injection at P13 or following anesthesia prior to imaging. Only live mice received fluorescein angiography. Out of the 24 mice that received FA imaging, 8 were PLGA, 8 were Beva and 8 were in the Free Beva group. Only clear and good quality images from 18 mice (6 PLGA-NP, 6 Beva-NP, and 6 Free Beva) were selected for QuantBV analysis of retinal vascular parameters. The images from the remaining 6 mice were excluded from retinal image analysis due to the development of cataracts or vitreous hemorrhage in one or both eyes during imaging (2 mice in the Beva group, 2 mice in the PLGA group, and 2 mice from the Free Beva group) which obstructed the retinal view and reduced the image quality.

## Conclusion

We optimized the fabrication of intravitreal Bevacizumab-loaded PVA-PLGA nanoparticles in mice with OIR. Treated right eyes showed anti-angiogenic activity with improved retinal artery tortuosity and retinal vein dilation, which signify severe ROP in infants. Nanoparticle delivery could alleviate concerns with repeat dosing and systemic toxicity seen with clinical use of Bevacizumab for the treatment of ROP and other Vaso proliferative diseases. Further studies are needed to investigate drug toxicity prior to clinical translation.

## Data Availability

The datasets generated and analyzed during the current study are available from the corresponding author on reasonable request.
